# The importance of family caregiving to achieving palliative care at home: a case report of end-of-life breast cancer in an area struck by the 2011 Fukushima nuclear crisis: A case report

**DOI:** 10.1097/MD.0000000000008721

**Published:** 2017-11-17

**Authors:** Akihiko Ozaki, Masaharu Tsubokura, Claire Leppold, Toyoaki Sawano, Manabu Tsukada, Tsuyoshi Nemoto, Kazuhiro Kosugi, Yoshitaka Nishikawa, Shigeaki Kato, Hiromichi Ohira

**Affiliations:** a Department of Surgery, Minamisoma Municipal General Hospital, Minamisoma, Fukushima, Japan; b Teikyo University Graduate School of Public Health, Tokyo, Japan; c Department of Internal Medicine, Soma Central Hospital, Soma, Fukushima, Japan; d Global Public Health Unit, School of Social and Political Science, University of Edinburgh, Edinburgh, UK; e Department of Research, Minamisoma Municipal General Hospital, Minamisoma, Fukushima, Japan; f Department of Home Medical Care, Minamisoma Municipal General Hospital, Minamisoma, Fukushima, Japan; g Department of Palliative Care, Kawasaki Municipal Ida Hospital, Kawasaki, Kanagawa, Japan; h Department of Health Informatics, School of Public Health, Kyoto University, Kyoto, Japan; i Research Institute of Innovative Medicine (RIIM), Tokiwa Foundation, Iwaki, Fukushima, Japan.

**Keywords:** bereavement, caregiver burden, caregiving, informal care, nuclear disaster, palliative care, social isolation

## Abstract

**Rationale:**

The primary setting of palliative care has shifted from inpatient care to patients’ residences. Family caregiving is essential for patients with life-limiting illnesses to receive palliative care at home, however little information is available regarding potential interventions to achieve palliative homecare for those without sufficient support from family members in various settings, including disasters.

**Patient concerns:**

In March 2011, Fukushima, Japan experienced an earthquake, tsunami and nuclear disaster. In August 2015, a 59-year-old Japanese female presented to our hospital, located 23 km north of Fukushima Daiichi Nuclear Power Plant, with a right breast ulcer.

**Diagnoses:**

The patient was diagnosed with stage IV breast cancer.

**Interventions:**

The patient's general condition gradually worsened despite a one-year course of chemotherapy, and she became bedridden after a fall in October 2016. Although the patient wished to receive palliative homecare, this appeared challenging to achieve because she resided alone in a temporary housing shelter. Although she originally lived with her family in Odaka District, Fukushima, she relocated outside of the city following evacuation orders after the disaster. The evacuation orders for Odaka District were still in effect when she returned to the city alone in 2014. We contacted her sister who moved apart from her during the evacuation, and explained the necessity of family caregiving to enable her palliative homecare.

**Outcomes:**

The sister decided to move back to their original residence in Odaka District and live with the patient again. The patient successfully spent her end-of-life period and died at home.

**Lessons:**

Health care providers and community health workers may need to take a pro-active approach to communicating with family members to draw informal support to enable patients’ end-of-life management according to their values and preferences. This is a lesson which may be applicable to broader healthcare settings beyond cancer, or disaster contexts, considering that population ageing and social isolation may continue to advance worldwide.

## 1 Introduction

Breast cancer is a significant part of the global burden of cancer, with a total of 571,000 deaths reported worldwide in 2015.^[1]^ However, the prognosis of patients with metastatic disease has gradually improved from a median overall survival of 15.7 months among those diagnosed from 1990 to 1992, to 25.2 months among those diagnosed from 2005 to 2012.^[2]^ Respect of individual values and preferences throughout the treatment course, while maximizing quality of life, is now regarded as an important part of the management of incurable patients.^[3]^

In the pursuit of patient-centered care delivery, the primary settings of end-of-life care have recently shifted from inpatient care to hospice centers and patient residences in high-income countries,^[4]^ and Japan is no exception.^[5]^ However, informal caregiving—care or assistance provided by any family member, relative, friend, or neighbor, based on a personal relationship with the patient—is essential for hospice or home-based care of the seriously-ill.^[6]^ Given the growing trends of aging, depopulation, and increasing numbers of people living alone or only with their spouses in Japan,^[7]^ end-stage breast cancer patients may be unable to obtain sufficient informal caregiving to receive palliative care at home. Furthermore, information is limited regarding possible ways to achieve palliative homecare among end-stage breast cancer patients in cases of compromised informal support.

**Figure 1:**
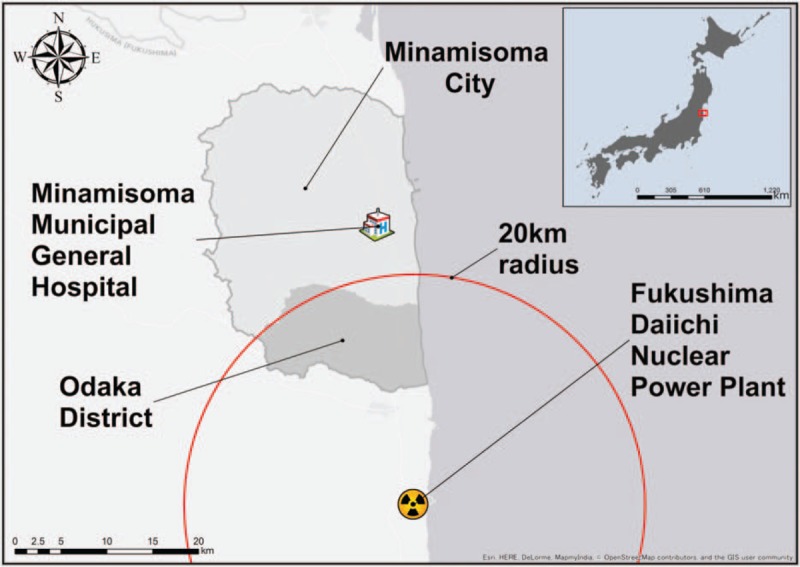
Geographical location of Minamisoma Municipal General Hospital and Odaka District.

In March 2011, an earthquake, followed by a tsunami and nuclear disaster at Fukushima Daiichi Nuclear Power Plant (FDNPP), struck the coastal area of Fukushima, Japan.^[8,9]^ Mandatory evacuation orders were originally issued for the 20 km radius of FDNPP and later expanded.^[8,9]^ However, many areas have had their evacuation orders lifted as of April 2017,^[10,11]^ and initiatives for the return of local residents have begun in affected areas, including Odaka District of Minamisoma City (Fig. 1).^[12]^ However, while evacuation orders were lifted for Odaka District in July 2016, its population remains at 1329 as of March 2017, a figure much lower than its original population of 12,842.^[13]^

As accentuated in Odaka District, nuclear disasters are a phenomenon which can deliver a devastating influence on the demographics of affected communities due to long-term evacuation and deterred restoration processes, possibly decreasing informal care available to affected residents with life-limiting illnesses.^[11,14]^ Here, we experienced a 59-year-old breast cancer patient who successfully received palliative homecare and died at her residence in Odaka District. This case represents the importance of family caregiving to achieve care in accordance with the individual patients’ values and preferences, and the role of health care providers to draw support from family members.

## 2 Case presentation

A 59-year-old Japanese female with no significant medical history presented to our hospital with a right breast ulcer with foul odor and bleeding in August 2015. She initially became aware of her breast lump in the summer of 2014, and the lesion started bleeding at the beginning of 2015. However, she did not recognize the seriousness of her symptoms, and delayed her first medical consultation for approximately 1 year. Pathological examination of the lesion confirmed a diagnosis of invasive breast cancer, and extensive imaging studies revealed multiple metastases of ipsilateral lymph node, lung, liver, and bone. She was clinically diagnosed with stage IV disease.

The patient originally lived with her mother, brother, and sister in Odaka District. Due to evacuation orders following the FDNPP accident, she relocated to a relative's residence 200 km away from the district, together with her brother and mother, while her sister evacuated separately from them. Although the patient moved back to Minamisoma City by herself in the middle of 2014, the mandatory evacuation orders issued to Odaka District continued to be in effect, and she began residing alone in one of the temporary housing shelters for evacuees built in the habitable zone of Minamisoma City. She reported that she did not frequently talk about her health concerns to her family or friends at this time, although she remained in general contact with them.

Despite inception of chemotherapy in September 2015, her general condition gradually deteriorated. In October 2016, she slipped and hit her pelvis on the floor, and became incapable of standing up. As a result, she was hospitalized. Although computed tomography (CT) did not reveal any fractures in her pelvis or right femur, she continued to be bedridden due to persistent pain, possibly caused by the metastatic lesion in her right femur, which was simultaneously detected by the CT. Her chemotherapy was discontinued. Although she disclosed her wish to die at home in November 2016, this appeared challenging to achieve because she was still residing alone in the confined conditions of the temporary housing shelter.

Our team then contacted her sister, who lived apart from the patient, and explained the necessity of family caregiving to enable her wish for palliative homecare.

As a result, the sister decided to not only live together with the patient, but also return to their prior residence in Odaka District. Although her condition had further deteriorated at the time of her discharge in December 2016, the patient did not report any concerns about moving back to her homeland.

A multidisciplinary team in our hospital, consisting of a home doctor, nurses, and a community health worker, provided her with health and social services (eg, rental service of nursing care bed) in Odaka District, where there were still no hospitals operating full-time since the disaster. In the course of palliative care management, we carefully listened to her sister, who was the primary caregiver for the patient, at every home visit, and gave her psychological support, and information about illness and services available to her. The patient did not make a single ER visit or hospital transfer, and died in January 2017 at her original residence in Odaka District. Bereavement support was not provided to the family members, including the patient's sister.

## 3 Discussion

This case suggests that health care providers and community health workers may be key players to successfully draw necessary resources for family caregiving, as seen here in the area struck by Japan's 2011 triple disaster, where significant demographic changes have occurred and medical systems remain vulnerable.

In our case, health care providers explained the importance of family caregiving to achieving the patient's wishes for palliative homecare to her sister, who lived separately from the patient post-disaster, and the sister decided to live together with the patient again and offer necessary support. In the coastal area of Fukushima, significant depopulation, aging of the remaining population, and the decreased numbers of family member per household have occurred following the 2011 FDNPP accident,^[11,15]^ and the potential for patients with significant medical needs to receive informal care at home may have decreased. Reduced chances to experience care at home may be particularly pronounced among those newly exposed to social isolation through post-disaster evacuation, as accentuated in our case. It has been demonstrated that social isolation itself delivers a detrimental effect on both physical and mental health in general settings,^[16]^ and we have previously found that this type of isolation can lead to delays in initial help-seeking behavior and limited treatment options, including end-of-life care, among cancer patients in disaster settings.^[17–19]^ However, there has been limited investigation on how to ameliorate the quantity and quality of the care among patients with life-limiting illnesses in limited health care resources, including a long-term aftermath of nuclear disasters. Here, we suggest that, to mitigate the health consequences induced by post-disaster social isolation and achieve end-of-life care following patient wishes, it may be necessary for health care providers in disaster-affected areas to intervene in patients’ social circumstances when necessary, beyond mere provision of medical services, which is the primary novelty of this report.

However, the potential for caregiver burden, an adverse effect of informal caregiving, should be noted particularly in the disaster-struck area of Fukushima, given that social isolation is an established risk factor in this region.^[20]^ It may have been fortunate that the period of caregiving was relatively short (1 month) in our case, as long-term caregiving is another risk factor for caregiver burden.^[20]^ Our consistent support toward the patient's sister may have additionally reduced the risk of caregiver burden.^[20]^ Yet, it should be noted that bereavement support for the family was not sufficiently discussed following our patient's death. Given that bereavement can be associated with negative outcomes in terms of physical and mental health among bereaved family members,^[21]^ provision of this type of support should have been at least taken into account in our case, as appropriate interventions, including psychosocial and psychological counseling programs, can improve health consequences among bereaved family members.^[21]^ While we suggest that it is imperative for health care providers to help achieve end-of-life care according to patients’ wishes, and reach out to family members as necessary, the potential for caregiver burden and bereavement, and ways to avoid their negative health consequences are also areas that deserve further consideration and discussion beyond this case report.

## 4 Conclusion

This is a case of a late-stage breast cancer patient who successfully received palliative homecare through family efforts and a multidisciplinary home medical care team in the disaster zone of Fukushima where medical systems remain vulnerable and families have been separated due to evacuation. It may be important for health care providers to draw necessary support from patients’ family members beyond the conventional framework of medical services, to achieve end-of-life care in accordance with patient values and wishes. Yet, a necessity of paying a sufficient attention to negative health outcomes of caregiver burden and bereavement should not be underrepresented. Given the global trend of population aging and an increasing prevalence of social isolation,^[22,23]^ our report suggests lessons which may be applicable to various health care settings beyond cancer or disaster contexts.
